# RAFI: Robust Authentication Framework for IoT-Based RFID Infrastructure

**DOI:** 10.3390/s22093110

**Published:** 2022-04-19

**Authors:** Vikas Kumar, Rahul Kumar, Akber Ali Khan, Vinod Kumar, Yu-Chi Chen, Chin-Chieh Chang

**Affiliations:** 1 Department of Mathematics, SSV College, Hapur 245101, Uttar Pradesh, India; vikas.chaudhary26@gmail.com (V.K.); ujjwalrahul@gmail.com (R.K.); 2 B. S. Anangpuria Institute of Technology and Management, Faridabad 121004, Haryana, India; cs.akberkhan@gmail.com or; 3 Department of Mathematics, PGDAV College, University of Delhi, New Delhi 110065, Delhi, India; vinod.iitkgp13@gmail.com or; 4 Department of Computer Science and Engineering, Yuan Ze University, Taoyuan 320, Taiwan; 5 Department of Computer Science and Information Engineering, National Taipei University of Technology, Taipei 106, Taiwan; 6 Department of Accounting Information, National Taipei University of Business, Taipei 100, Taiwan; ccchang@ntub.edu.tw

**Keywords:** IoT, RFID, security, authentication, random oracle model

## Abstract

The Internet of Things (IoT) is a future trend that uses the Internet to connect a variety of physical things with the cyber world. IoT technology is rapidly evolving, and it will soon have a significant impact on our daily lives. While the growing number of linked IoT devices makes our daily lives easier, it also puts our personal data at risk. In IoT applications, Radio Frequency Identification (RFID) helps in the automatic identification of linked devices, and the dataflow of the system forms a symmetry in communication between the tags and the readers. However, the security and privacy of RFID-tag-connected devices are the key concerns. The communication link is thought to be wireless or insecure, making the RFID system open to several known threats. In order to address these security issues, we propose a robust authentication framework for IoT-based RFID infrastructure. We use formal security analysis in the random oracle model, as well as information analysis to support the claim of secure communication. Regarding the desirable performance characteristics, we describe and analyze the proposed framework’s performance and compare it to similar systems. According to our findings, the proposed framework satisfies all security requirements while also improving the communication.

## 1. Introduction

An RFID infrastructure has a symmetric nature. The RFID system is a wireless technology that is used to identify remote objects that have RFID tags embedded in them. RFID technology is utilized in a variety of applications, including transportation, supply chain management, livestock management, e-passport, e-payment, and patient healthcare [1,2,3]. Backend readers, servers, and tags are all a part of a conventional RFID system whose architecture is symmetric, since the dataflow is in one direction from the tag, reader to server, and then, the inverse Table 5. The lack of physical contact between the reader and the tags is a crucial element of RFID systems, and the following are some of the benefits of using them: RFID tags are small and inexpensive, and radio frequency communication can recognize large numbers of RFID tags at the same time [4,5]. RFID systems, on the other hand, are exposed to a variety of security attacks and privacy exposure concerns due to their use of wireless communication and signal broadcasting techniques. It is difficult to apply a comprehensive cryptographic algorithm to an RFID system due to the strictly limited calculation resources, tiny storage capacity, and weak power supply of low-cost tags, and these issues are impeding the rapid development of this technology [6]. RFID security is fundamentally concerned with authentication and privacy issues. A secure protocol running RFID tags and readers can provide authentication. If a tag contains unique secret information and the RFID reader and RFID tag can convince the RFID reader that they both have that information, the tagged product is considered to be authentic and the person has access to it. Tag anonymity is one of the most important features that any RFID-based authentication technique aspires to attain, and tag untraceability, which ensures the privacy of the tag or the mobility of a user wearing an RFID tag, is a more satisfactory property of tag anonymity. To achieve this attribute, a tag must encode its original identity using a cryptographic primitive such as a one-way secure collision-resistant hash function in existing state-of-the-art authentication protocols. RFID is the simplest form of pervasive sensor network and is widely used for object identification [7]. RFID systems are made up of a tag with a transceiver that sends and receives radio signals from connected devices [8,9]. The RFID reader is another device that acts as an access point and can receive and deliver messages to transceivers. The reader is also in charge of ensuring that tag information is available at the application level [10]. IoT-based RFID tags can be of the passive or active type. The differences between these tags are summarized in Table 1.

### 1.1. Related Work

In recent years, numerous exciting anonymous IoT-based RFID authentication and key agreement frameworks have been proposed, which can be classified into Public Key Cryptosystem- (PKC) and Non-Public Key Cryptosystem- (NPKC) based authenticated schemes. These approaches are unsuitable for tiny powered tags due to the modular exponential operations. Hash-based RFID systems, on the other hand, would be the best choice among NPKCs because of their low computational overhead [7,11,12,13]. Yang et al. [11] introduced an authentication mechanism based on a one-way secure collision-resistant hash function and exclusive-OR, claiming that it addressed all of the security vulnerabilities that occur in RFID systems. Unfortunately, the protocol is vulnerable to many attacks, including “man-in-the-middle”, forgeries, and loss of untraceability [14]. Cho et al. [13] developed a secure hash-based authentication framework, claiming that it addresses all of the security, privacy, and forgery difficulties that exist in RFID communication systems. However, Safkhani et al. [15] recently demonstrated that the protocol does not meet the authors’ security promises. In their paper, they cryptanalyzed Cho et al.’s [13] protocol and concluded that it is vulnerable to “de-synchronization or DoS attacks, tag impersonation attacks, and reader impersonation attacks”. Furthermore, they showed in their paper that all proposed lightweight authentication techniques based on one-way hash functions and exclusive-OR are impracticable [11,12,13,16,17]. Ayaz et al. [18] suggested another mutual authentication approach for secure RFID communication systems utilizing only symmetric key cryptography operations. In this framework, an authentication is accomplished on the basis of user biometrics’ verification in their protocol. Liu et al. [19] proposed an authentication protocol for an RFID system by using hash and XoR operations. The correctness of the protocol was proven by using “Burrows–Abadi–Needham (BAN)” logic analysis. Mansoor et al. [20] proposed a securing IoT-based authentication protocol for RFID systems by using a symmetric cryptography approach. Unfortunately, we studied their protocol and found the security weaknesses of their protocol. Furthermore, Mansoor et al. [20] showed that the protocol proposed by Gope et al. [21] is vulnerable to collision attacks, DoS attacks, and stolen verifier attacks. In 2022, Gao and Lu pretested a new ultra-lightweight RFID authentication protocol in passive RFID systems [22]. The proposed protocol, they claimed, prevents numerous known attacks, beats several existing ultra-lightweight protocols in terms of computational cost, storage requirements, and communication costs, and is efficient in terms of the computational cost, storage requirements, and communication costs. Wang et al. suggested a protocol [23] for which they had formal and informal discussions about security and privacy. Xiaomei et al. discussed [24] the RFID logic of an event-based authentication framework for secure communication. Shariq et al. proposed an RFID-based anonymous and secure framework for deployment in IVs [25]. Wei et al. proposed an improved security authentication protocol for lightweight RFID based on ECC [26]. Arslan and Bingöl presented the security and privacy analysis of recently proposed ECC-based RFID authentication schemes [27].

### 1.2. Adversary Model 

Our adversary model is based on the threat model of [28], which is well-known and widely recognized. By altering, monitoring, deciding on, and introducing information into the communications channel, the attacker can not only see the communications channel, but also capture session keys, confidential documents, and private keys stored in the contributor memory through explicit attacks. Many assaults, such as replay attacks, man-in-the-middle attacks, impersonation attacks, etc., are now possible in the RFID system due to the utilization of public communication networks and wireless communication networks. As a result, the privacy and security issues are major concerns in RFID frameworks. Thus, an authentication and key management mechanism is required to validate the legitimacy of specified entities.

### 1.3. Security Requirements for an IoT-Based RFID Communication System

As far as we know and based on the available literature, many authentication protocols for RFID communication systems have been presented during the last few years. In RFID systems, authentication and key agreement are the best approaches to make them suitable for a wide range of applications. During the transmission of messages between RFID tags and RFID readers, many types of security attacks may occur. We outline various security needs in light of these issues, such as forward security, mutual authentication, anonymity, scalability, confidentiality, untraceability,“ man-in-the-middle attack, insider attack, replay attack, impersonation attack”, etc., to provide secure communication for the RFID system. Such requirements are utilized as the criteria for assessing the RFID system in order to provide a secure and efficient authentication protocol. The following security criteria should be met by any authentication scheme that attempts to secure a practical RFID-based system:

- Mutual authentication: This is the most important aspect of any authentication mechanism. Furthermore, mutual authentication must be achieved in the presence of all three RFID system participants. The authentication process takes place between the backend database server and the RFID tag. Messages are sent between the tag, reader, and server over an unsecured communication channel.
- Tag anonymity: To minimize forgery and ensure security, this is the most important and necessary security requirement. Furthermore, if an opponent is unable to trace an RFID tag during message delivery over a public channel, the RFID authentication system maintains its anonymity. Anonymity can be divided into two categories: strong anonymity and weak anonymity. Furthermore, in IoT communication, the participants involved do not disclose their real identity in order to defend their security and privacy.
- Message authentication: In Internet operations, this maintains the integrity of message communication.
- Untraceability: In the RFID communication system, untraceability means that no one can trace the behavior patterns of the participants involved and their forwarded messages.
- Session key agreement: Following the successful implementation of the proposed protocol, a session key agreement will be established between users with their mobile devices and the network control center for future communication.
- Confidentiality: Encrypting shared secrets on the public channel ensures the security of RFID communications between the tag and reader.
- Perfect forward secrecy: Perfect forward secrecy is a technique that should be used in the authentication protocol design to give secrecy to previously communicated messages, where an opponent who discovers the entities private and public keys will be unable to derive a past session key.
- Scalability: The approach is not scalable if the server conducts an extensive search to verify a tag. Worse, an opponent may conduct a timing attack [29] against the protocol, which can identify a tag based on how long it took the server to authenticate it. To maintain scalability, an authentication strategy should avoid any exhaustive search operations.
- Availability: In an RFID system, the authentication and key agreement procedure runs all the time between the RFID tag and RFID backend database server. In most authentication methods, the shared secret information between the RFID tag and RFID backend database server must be updated to achieve the attribute of accessibility. However, security risks such as Denial-Of-Service (DoS) or de-synchronization attacks may disrupt this process. The RFID system’s efficiency may be harmed as a result of these concerns. Thus, when designing an authentication protocol, this issue should be considered.
- Impersonation attack: An adversary could try to mimic legitimate protocol participants (such as the cloud database server, RFID reader, or RFID tag) by replaying a message captured from the channels. Any impersonation should be avoided at all costs.
- Replay attack: An outsider attempts to confuse other certified participants by restating intercepted data in this attack. This attack targets a user whose information is intercepted by an uncertified third party.
- Man-in-the-middle attack: An adversary listens in on transmitted data and then attempts to delete or manipulate the contents of the data sent to receivers in this attack.
- Insider attack: Any insider can play the role of adversary in the RFID communication system.
- De-synchronization attack: An adversary may generate desynchronization problems if a protocol authentication is based on shared values. The server may be unable to verify the tag in the future if the shared data are updated by the server, but the tag is not. De-synchronization attempts should be avoided.

### 1.4. Motivation and Contribution

Many authentication and key agreement frameworks for RFID systems have been presented during the last few decades, as far as we know and based on the existing literature [13,16,17,19,20,21]. However, a suitable authenticated key agreement protocol for RFID systems that is secure and efficient for RFID systems is missing. RFID systems require an authenticated key agreement scheme because of their varying computing capabilities and privacy requirements. Thus, we propose an authenticated key agreement protocol for RFID communication systems. Table 2 shows the comparative study of the advantages and disadvantages of other protocols with respect to our suggested protocol. The following are some notable characteristics of the proposed framework:

- We propose a robust authentication protocol that supports key agreement between RFID tags and the database server for IoT-based RFID infrastructure.
- We give a thorough explanation of the informal security study, proving that the suggested protocol can resist a variety of well-known security attacks.
- The proposed protocol security is formally demonstrated using a random oracle model.
- The proposed the RAFI has desirable security features that make the proposed protocol robust and efficient, according to the proof of security.
- The results of the performance evaluation and comparison show that the proposed RAFI has desirable performance features.

**Table 2:** Merits and demerits of the existing authentication protocols in RFID environments.

Protocols	Approach Used	Published Year	Merits	Demerits
Tan et al. [16]	Hash function	2008	Provides backward and forward secrecy	Susceptible to replay attack, insider attack,
			and de-synchronization	DoS attack, and tag anonymity problem
Cai et al. [17]	Hash function	2009	Provides a mutual authentication and	Vulnerable to impersonation attack,
			anonymity and secure against stolen verifier attack	insider attack, and DoS attack
Cho et al. [13]	Hash function	2015	Provides a mutual authentication and tag untraceability	Prone to insider attack, man-in-the-middle attack
			and secure against stolen verifier attacks	and impersonation attack
Gope and Hwang [21]	Hash function	2015	Prevents replay attacks, de-synchronization,	Vulnerable to collision attacks,
			and man-in-the-middle attack	DoS attacks, and impersonation attack
Liu et al. [19]	Hash function	2018	Provides mutual authentication,	Susceptible to stolen verifier attacks,
			tag untraceability, and tag anonymity	collision attacks, and DoS attacks
Mansoor et al. [20]	Hash function	2019	Attains mutual authentication, scalability,	Vulnerable to impersonation attack, man-in-
			and data confidentiality	the-middle attack, collision attack, and replay attack

### 1.5. Organization of the Paper

The remainder of the proposed framework is organized as follows: Section 2 covers the fundamentals of the mathematics. The proposed framework is discussed in Section 3. In Section 4, the proposed framework security is evaluated. Section 5 includes a performance study of the proposed framework. Finally, the findings are summarized in the Section 5.4.

## 2. Mathematical Preliminaries

The notations and terminology used in the RAFI are defined in this section.

### 2.1. Notations

As shown in Table 3, the following notations are utilized.

### 2.2. Cryptography Materials

Here, various cryptographic primitives that are used to design the proposed security protocol are discussed. In this regard, we make use of lightweight cryptographic primitives to ensure security and computational efficiency.

#### 2.2.1. Cryptographic Hash Function

The hash operation takes a variable-length message (M) as the input and outputs a fixed string result H(M), which is known as the message digest. In practice, reversing this process is nearly impossible. As a result, this function is referred to as a collision-resistant one-way hash function. Following that, our system integrity will be protected using the Secure Hash Algorithm (SHA-256). The one-way collision-resistant $h:{\left\{0,1\right\}}^{*}\to {\left\{0,1\right\}}^{n}$ hash function [30,31,32] takes an input $x\in {\left\{0,1\right\}}^{*}$ and returns an output $h\left(x\right)\in {\left\{0,1\right\}}^{n}$ of definite length n of a message. The advantage of any A for calculating the collision is as follows: Advantage $Adv_{A}^{HASH}\left(t\right)=Pr[\left(x_{1},x_{2}\right){⇐}_{R}A:x_{1}\neq x_{2}$, and $h\left(x_{1}\right)=h\left(x_{2}\right)]$ and $\left(x_{1},x_{2}\right){⇐}_{R}A$ represent the set of $\left(x_{1},x_{2}\right)$ computedby attacker A. The probability of this advantage is thus calculated across the random choice values made by A with the run duration t. Hash function h(.) is collision-resistant if $Adv_{A}^{HASH}\left(t\right)\leq \epsilon$, where ϵ>0.

#### 2.2.2. XoR Cipher

In cryptography, the XoR operation includes some postulates: P⊕(Q⊕R)=(P⊕Q)⊕R, P⊕P=0, P⊕0=P, and (Q⊕P)⊕P=Q⊕0=Q.

## 3. The Proposed Protocol

**Figure 1:**
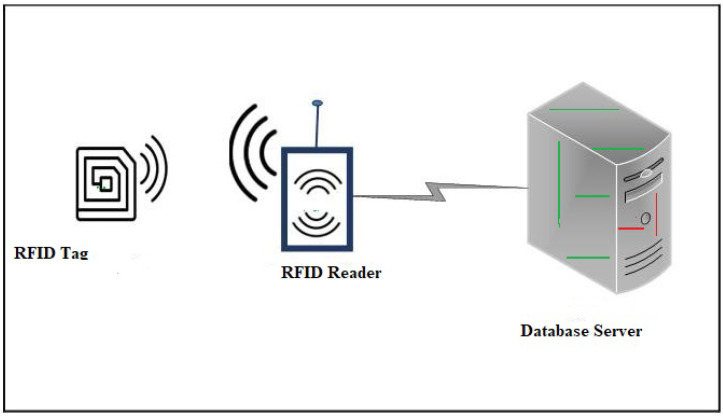
Architecture of the RAFI.

The steps in the proposed framework are as follows: “ registration phase of RFID with database server” and “login and authentication phase”. The architecture of the proposed protocol given the Figure 1.

### 3.1. Registration Phase

The following are the instructions for registering the RFID tag with the database server. The detailed of this phase also mentioned in Table 4.

Step AK1: To register with database server *S*, tag $T_{i}$ inputs $ID_{T_{i}}$ and, then, $T_{i}\Rightarrow S:M_{R_{i1}}=\left\{ID_{T_{i}}\right\}$ via a secure channel.
Step AK2: Upon receiving $M_{i1}$, it generates sequence number $SN_{i}$ for $T_{i}$ and computes $S_{1}=ID_{S}⊕h(ID_{T_{i}}∥SN_{i}∥x_{S})$ where $x_{S}$ is private key for *S*. Furthermore, the data server computes $S_{2}=h\left(S_{1}∥ID_{T_{i}}\right)⊕ID_{T_{i}}$. Finally, *S* stores $S_{1},S_{2},SN_{i}$ in the database and sends $M_{R2_{i2}}=\left\{S_{1},S_{2},SN_{i},h\left(.\right)\right\}$ towards the tag via a secure medium.
Step AK3: Upon receiving $M_{R2_{i2}}$, the RFID tag stores parameters $\left\{S_{1},S_{2},SN_{i}\right\}$ in the database for further communication via a secure medium.

### 3.2. Login and Authentication Phase

$T_{i}$ successfully registers with *S*, and when she/he wants to use the service, she/he makes an access request to *S*. The following is a description of the procedure in steps. Further, The detailed of this phase also mentioned in Table 5.

Step MA1: $T_{i}$ generates random value *r* and computes the following values $r_{1}=r⊕\left(S_{1}⊕S_{2}\right)$, $H_{1}=h(ID_{T_{i}}∥S_{1}∥S_{2})$, $H_{1}^{1}=H_{1}⊕S_{2}$. Furthermore, $T_{i}$$\to R_{j}:M_{1}=\left\{r_{1},H_{1}^{1},T_{1}\right\}$.
Step MA2: Upon receiving $M_{1}$, RFID reader $R_{j}$ verifies $T_{2}-T_{1}\leq ▵T$ and $R_{j}$$\to S:M_{2}=\left\{r_{1},H_{1}^{1},T_{3}\right\}$.
Step MA3: Upon receiving $M_{2}$, S verifies $T_{4}-T_{3}\leq ▵T$. Then, S computes $H_{1}^{*}=H_{1}⊕S_{2}$ and verifies $H_{1}^{*}\overset{?}{=}H_{1}^{1}$; if this condition does not hold, then it terminates the process; otherwise, S computes $r^{*}=r_{1}⊕\left(S_{1}⊕S_{2}\right)$, generates a random value $r_{2}$, computes the link of computations $SK_{S}=h(ID_{S}∥ID_{T_{i}}∥r^{*}∥r_{2}∥SN_{i}∥S_{1}∥S_{2}∥T_{5})$, $H_{2}=h(S_{1}∥S_{2}∥r^{*})$, $H_{2}^{2}=H_{2}⊕\left(r^{*}⊕S_{2}\right)$, $K_{1}=ID_{T_{i}}⊕h(r^{*}∥SN_{i}∥H_{1}^{*})$, and encrypts $E_{1}=E_{K_{1}}\left(H_{2}^{2},r_{2},ID_{S},T_{5}\right)$. Finally, $S\to R_{j}:M_{3}=\left\{E_{1},T_{5}\right\}$.
Step MA4: Upon receiving $M_{3}$, $R_{j}$ verifies $T_{6}-T_{5}\leq ▵T$. Furthermore, $R_{j}\to T_{i}:M_{4}=\left\{M_{3},T_{7}\right\}$.
Step MA5: Upon receiving $M_{4}$, $T_{i}$ verifies $T_{8}-T_{7}\leq ▵T$ and decrypts $\left(H_{2}^{2},r_{2},ID_{S},T_{5}\right)=D_{K_{2}}\left(E_{1}\right)$ with the help of computed key $K_{2}=ID_{T_{i}}⊕h(r∥SN_{i}∥H_{1})$. Furthermore, it computes $H_{2}^{*}=H_{2}^{2}⊕\left(r⊕S_{2}\right)$ and verifies $H_{2}^{*}\overset{?}{=}H_{2}$. Finally, Tag sets the session key for furter communication as $SK_{T}=h(ID_{S}∥ID_{T_{i}}∥r∥r_{2}∥SN_{i}∥S_{1}∥S_{2}∥T_{5})$. Hence, session key agreement $SK=SK_{T}=SK_{S}$.

## 4. Security Analysis

The security analysis of the proposed protocol is conducted by a formal method and an informal method as follows.

### 4.1. Informal Security Analysis

The following is an informal security analysis of the proposed protocol.

#### 4.1.1. Key Freshness

In the proposed protocol, the session key contains the timestamp and a freshly generated random number. Furthermore, in the authentication procedure, the timestamp and random number are distinct for each session. The uniqueness of these parameters confirms the session’s unique key. Thus, the unique key for each session confirms the key freshness property of the proposed protocol.

#### 4.1.2. Untraceability

If a cryptographic scheme has two features, it is untraceable. A is unable to distinguish between users’ initial identities; A is unable to determine whether two distinct sessions starting at different times belong to the same user. Thus, it is intended that both properties be maintained.

#### 4.1.3. Session Key Agreement

In the proposed scheme, the database server calculates $SK_{S}=h(ID_{S}∥ID_{T_{i}}∥r^{*}∥r_{2}∥SN_{i}∥$$S_{1}∥S_{2}∥T_{5})$ and the RFID tag computes $SK_{T}=h(ID_{S}∥ID_{T_{i}}$$∥r∥r_{2}∥SN_{i}∥S_{1}∥S_{2}∥T_{5})$. Thus, $SK_{S}=SK_{T}$. Thus, the proposed protocol maintains the said cryptographic property.

#### 4.1.4. Session Key Verification

The RFID tag verifies its session key in our proposed system as $H_{2}^{*}\overset{?}{=}H_{2}$, where $H_{2}^{*}=H_{2}^{2}⊕\left(r⊕S_{2}\right)$ and $H_{2}^{2}=H_{2}⊕\left(r^{*}⊕S_{2}\right)$, embedded with many secret credentials. Therefore, the proposed technique allows for the verification of session keys.

#### 4.1.5. Scalability

In the proposed protocol for the RFID system, the RFID server S does not perform an exhaustive process to authenticate each RFID tag. The RFID server S, on the other hand, validates the RFID tag and reacts immediately to it. This increases the scalability of the proposed protocol.

#### 4.1.6. Forward Secrecy

Given that the proposed protocol only uses symmetric key cryptography, i.e., the secure collision-resistant hash function, and we do not update the shared parameters per session, it is not possible to give this property, similar to any other protocol in this context. It should be emphasized that if the protocol employs a public key primitive, this attribute can be simply provided.

#### 4.1.7. Traceability and Anonymity

In the proposed protocol, the exchanged messages are $M_{1}$ and $M_{2}$. In these messages, excluding $T_{i}$ and $T_{j}$, which are the timestamps and cannot be connected to any identity to trace or compromise its anonymity, the rest of the information is encrypted values or the output of the one-way hash function and from one session to another session is randomized by fresh nonce values. Hence, the exchanged messages do not reveal any information to trace the tag or server or compromise their anonymity.

#### 4.1.8. Replay Attack

Random numbers and timestamps are common countermeasures in replay attacks. However, in the proposed protocol, both of them are present. The timestamp condition checks $T_{i}-T_{j}\leq △T$, where △T is the valid period, and $a,b\in Z_{q}^{*}$, where *a*, *b* are fresh random numbers and *q* is a large prime number.

#### 4.1.9. Privileged Insider Attack

In the proposed protocol, interacting participants and a third party do not maintain any verifier repository. The authentication procedure is performed by participants using their unique secret keys. Thus, the proposed protocol resiststhe stolen verifier and insider threats.

#### 4.1.10. Man-in-the-Middle Attack

The protocol is secure against the man-in-the-middle attack. The adversary is not successful in obtaining the key and pseudonym value. Furthermore, hash functions ensure message integrity, and timestamps control the session time; therefore, any message modification or unexpected delay by a “man-in-the-middle attack” will be detected with a high probability. In the proposed protocol, we verify conditions on both sides, $H_{1}^{*}\overset{?}{=}H_{1}$ and $H_{2}^{*}\overset{?}{=}H_{2}$. As a result, the proposed protocol is protected from the “man-in-the-middle attack”.

#### 4.1.11. Impersonation Attack

To impersonate the RFID tag, the attacker should either perform a replay attack or generate a valid $M_{1}$. However, the replay attack is not feasible in this proposed protocol, and the attacker also has no chance to compute a valid $M_{1}$, because it does not have access to $SK_{i}$. The same logic can be applied to an impersonating server. Hence, the proposed framework is safe from impersonation attacks.

#### 4.1.12. De-Synchronization Attack

There is no secret sharing between the RFID tags and the RFIF backend server in the proposed protocol. Furthermore, no value needs to be updated in each authentication session. Thus, our suggested protocol is resistant to the de-synchronization attack.

#### 4.1.13. Parallel Session Attack

When an A reprocesses past messages in an insecure channel to compose a new request, this is known as a parallel session attack. To retrieve the key, A impersonates the user tag $T_{i}$. The secret credentials, which are used to compute the content, must be known by A before user $T_{i}$ may compute a valid login request or execute the session key. It is apparent from the preceding study that A is unable to obtain the session key. Hence, the proposed framework protects against the parallel session attack.

### 4.2. Formal Security Analysis

In this section, the random oracle model is deployed to demonstrate that the beacons exchanged in the proposed protocol are robust against any form of eavesdropping, and hence, the communicating entities can trust each other as they communicate over insecure channels.

#### 4.2.1. Handshake Model

The handshake stage is used to exchange information and perform device synchronization amongst the participants. This is also the point at which the server takes control of the process and maintains it until the user is authenticated. At this level, the input is in the form of a classical medium, but the output is in the form of a quantum medium. The handshake stage is used to exchange information and perform device synchronization amongst the participants. This is also the point at which the server takes control of the process and maintains it until the user is authenticated. At this level, the input is in the form of a classical medium, but the output is in the form of a quantum medium. The handshake authentication model for the proposed RFID protocol shown in the Table 6.

#### 4.2.2. Formal Security Model

The formal model for the propose framework, which is based on the random oracle model, is discussed in this section [33,34]. We made some changes to the original to make it work with the proposed framework. We employed three participants to demonstrate our proof, T,R, and *S* as the RFID tag, the RFID reader, and the database server. $ID_{T_{i}}$ is the identity of *T*. Similarly, $ID_{S}$ is the identity of *S*. N is the identities’ dictionary. More information about this model may be found in [35].

#### 4.2.3. Formal Security Proof

In this part, we show the proposed framework’s formal security using a model [28] based on the random oracle model [33,34]. In this model, an adversary A can interact with framework entities, say Ω, which is a server.

**Theorem** **1.** *Suppose that A is a polynomial-time attacker attempting to compromise the protocol semantic security and close to the $Q_{H}$ hash query, $Q_{e}$ execute query, $Q_{s}$ send query, $Adv_{E_{K}}^{SE}\left(A\right)$ is the advantage of A, and |D| is the set of uniformly distributed cardinality. Thus, the advantage of A in the proposed protocol is given by*

$$\begin{array}{c} Adv_{rfid}\left(A\right)\leq \frac{\left(Q_{H}^{2}+Q_{S}\right)}{2^{L-1}}+\frac{{\left(Q_{S}+Q_{E}\right)}^{2}}{p}+\frac{2Q_{S}}{\left|D\right|}+2Adv_{E_{K}}^{SE}\left(A\right) \end{array}$$

**Proof.**  For the proof of this theorem, we introduce the game of series, initially with *GM*_0_ the real attack, and stop with *GM*_5_ where A has no advantage. The details of these are explained as below in *GM*_0_ to *GM*_5_. Further, the simulation queries based on this random oracle model are ginen in Table 7.

*GM*_0_: The execution of *Game* *GM*_0_ is the same as the real attack in the oracle model. We have
  (1) $$\begin{array}{c} Adv_{rfid}\left(A\right)=\left|2Pr\left[Succ_{0}\right]-1\right|. \end{array}$$
*GM*_1_: Different queries are conducted in *GM*_1_, and the results of the queries are kept in the oracle lists, making it impossible for an attacker to distinguish between the two oracle games. As a result, we have
  (2) $$\begin{array}{c} Pr\left[Succ_{1}\right]=Pr\left[Succ_{0}\right]. \end{array}$$
*GM*_2_: The execution of *GM*_2_ is like *GM*_1_, except that *GM*_2_ stops when a collision is present in the hash function and information messages. Therefore, the birth day paradox, the probability of collision in the transcript is $\frac{{\left(Q_{S}+Q_{E}\right)}^{2}}{2p}$ at most [36], and the success probability of secure hash function collision is at most $\frac{Q_{H}^{2}}{2^{L+1}}$. Hence, we have
  (3) $$|Pr\left[Succ_{2}\right]-Pr\left[Succ_{1}\right]|\leq \frac{Q_{H}^{2}}{2^{L+1}}+\frac{{\left(Q_{S}+Q_{E}\right)}^{2}}{2p}.$$
*GM*_3_: The simulation of $GM_{3}$ is identical to that of $GM_{2}$, with the exception that $GM_{3}$ will be terminated if A guesses the verifier operations without knowing the random oracle. Until the server grid fails in a legitimate authentication request, $GM_{3}$ and the preceding game are different. As a result, we have
  (4) $$|Pr\left[Succ_{3}\right]-Pr\left[Succ_{2}\right]|\leq \frac{Q_{S}}{2^{L}}$$
*GM*_4_: $GM_{4}$ is the same as $GM_{3}$, except that only the test inquiry of $GM_{4}$ stops when adversary A discloses a TestID to obtain the real identity $ID_{i}$ or sends a query to obtain the password information. Therefore, we conclude that
  (5) $$|Pr\left[Succ_{5}\right]-Pr\left[Succ_{4}\right]|\leq \frac{Q_{S}}{\left|D\right|}+Adv_{E_{K}}^{SE}\left(A\right).$$
*GM*_5_: The execution of $GM_{5}$ is the same as $GM_{4}$, except that only TestSK of $GM_{5}$ will stop when adversary A publishes a secure hash inquiry with $h(ID_{S}∥ID_{T_{i}}∥r∥r_{2}∥SN_{i}∥S_{1}∥S_{2}∥T_{5})$, because A by utilizing the secure hash inquiry obtains the SK with success probability $Q_{H}^{2}/2^{L+1}$. Therefore, we have
  (6) $$|Pr\left[Succ_{6}\right]-Pr\left[Succ_{5}\right]|\leq \frac{Q_{H}^{2}}{2^{L+1}}$$
         Thus, A does not contain a favorable advantage in perceiving the actual SK from an arbitrary random one without making a hash query with the true input, $Pr[Succ_{6}]=1/2$. Adding every one of these probabilities, we can conclude that the theorem is proven.

□

**Table 7:** Simulation of oracles.

Simulation Queries
Hash queries $h_{n}\left(m\right)$, *n* = 0, 1, 2, 3, 4, 5. If $\left(m,hv_{n}\right)$ exists in the index list of $L_{h_{n}}$, the value $hv_{n}$ will be returned. Otherwise, the generated random value will be added to the index list $L_{h_{n}}$.
Computes $r_{1}=r⊕\left(S_{1}⊕S_{2}\right)$
Computes $H_{1}=h\left(ID_{T_{i}}S_{1}∥S_{2}\right)$
Computes $H_{1}^{1}=H_{1}⊕S_{2}$
Then, it answers with $M_{1}=\left\{r_{1},H_{1}^{1},T_{1}\right\}$
For the $send(V,\left\{r_{1},H_{1}^{1},T_{1}\right\}$ query, the G oracle simulates the following steps:
Verifies $T_{2}-T_{1}\leq ▵T$
Then, it answers with $M_{2}=\left\{r_{1},H_{1}^{1},T_{3}\right\}$
For $send(G,\left\{r_{1},H_{1}^{1},T_{3}\right\}$ query, the V oracle simulates the following steps:
Computes $H_{1}^{*}=H_{1}⊕S_{2}$
Verifies $H_{1}^{*}\overset{?}{=}H_{1}$
Computes $r^{*}=r_{1}⊕\left(S_{1}⊕S_{2}\right)$
Generates random value $r_{2}$
Computes $SK_{S}=h(ID_{S}∥ID_{T_{i}}∥r^{*}∥r_{2}∥SN_{i}∥S_{1}∥S_{2}∥T_{5})$
Computes $H_{2}^{2}=H_{2}⊕\left(r^{*}⊕S_{2}\right)$
Computes $K_{1}=ID_{T_{i}}h(r^{*}∥SN_{i}∥H_{1}^{*})$
Encrypts $E_{1}=E_{K1}\left(H_{2}^{2},r_{2},ID_{S},T_{S}\right)$
Then, it answers with $M_{3}=\left\{E_{1},T_{5}\right\}$
For the $send(V,\left\{E_{1},T_{5}\right\}$ query, the oracle simulate the following steps
Verifies $T_{6}-T_{5}\leq ▵T$
Then, it answer with $M_{4}=\left\{M_{3},T_{7}\right\}$
For $send(G,\left\{M_{3},T_{7}\right\}$ query, the T oracle simulates the following steps:
Verifies $T_{8}-T_{7}\leq ▵T$
Computes $K_{2}=ID_{T_{i}}h(r∥SN_{i}∥H_{1})$
Decrypts $\left(H_{2}^{2},r_{2},ID_{S},T_{S}\right)=D_{K2}\left(E_{1}\right)$
Computes $H_{2}^{*}=H_{2}^{2}⊕\left(r⊕S_{2}\right)$
Verifies $H_{2}^{*}\overset{?}{=}H_{2}$
Computes $SK_{T}=h(ID_{S}∥ID_{T_{i}}∥r∥r_{2}∥SN_{i}∥S_{1}∥S_{2}∥T_{5})$
For an Execute $\left(T^{i},R^{t},S^{j}\right)$ query, all Send queries are consecutively completed.
Massage $\left(M_{1},M_{2},M_{3},M_{4}\right)$ is the output.
For a $Reveal\;(I^{K})$ query, if the chance $I^{K}$ has been settled and provided a safe session key, output $SK_{T}$ or $SK_{S}$; otherwise, ⊥ is the response.
For a $Corrupt\;(I^{K})$ query, all the information of $I^{K}$ is returned.
For a $Test\;(I^{K})$ query, if $I^{K}$ is not fresh, return ⊥; otherwise, a coin γ is tossed.
If γ=0, the output is a random value with length *l*.
If γ=1, the conclusion is the appropriate session key.

## 5. Performance Analysis

The performance analysis of the proposed framework compared to related frameworks [13,16,17,19,20,21] is given in three subsections: comparison of the security and functionality features and the computational and communication cost comparisons. The conclusion of the performance analysis demonstrates that the proposed framework has better efficiency and security in RFID communication systems.

### 5.1. Comparison of the Security and Functionality Features

The features that an authentication protocol is supposed to have are known as security requirements. These properties or needs must be guaranteed by every authentication protocol. The suggested protocol was compared to current protocols based on these requirements. The features/requirements examined for the comparison analysis are listed below.

In Table 8, we summarize the security properties of the proposed framework and those schemes that are available in literature [13,16,17,19,20,21]. The related schemes can be seen with different security shortcomings against various security attacks.

### 5.2. Comparison of the Computational Cost

We calculated the computational cost of the RAFI and compared it to other frameworks [13,16,17,19,20,21], which is illustrated in Table 9. The computation time of the execution of hash operation ($T_{h}$) was 0.0023 ms, while the computation time of the execution of the encryption and decryption ($T_{E/D}$) was 0.0046 ms. The experiment was conducted on an Ubuntu system with a 2.20 GHz Intel dual-core Pentium CPU with a 2048 MB processor and RAM [20,37].

The protocol presented in [16] incurred 2$T_{h}$, 2$T_{h}$, and 3$T_{h}$ for each RFID tag, RFID reader, and database server, respectively, and the total computational cost in their protocol was 4$T_{h}\approx 0.0161$. In the same way, the protocols’ computational cost was provided in [17] to be 4$T_{h}$, 2$T_{h}$, and 6$T_{h}$ for each RFID tag, RFID reader, and database server, respectively, for each participant, totaling 12$T_{h}\approx 0.0276$. The computational cost presented in [13] was 3$T_{h}$, 2$T_{h}$, and 5$T_{h}$ for each participant, totaling 10$T_{h}\approx 0.023$. The computational cost in [21] was 5$T_{h}$ for the RFID tag, 2$T_{h}$ for the reader, and 7$T_{h}$ for the database serve; therefore, the total computational cost in their framework was 14$T_{h}\approx 0.0322$. The computational cost in [19] for the RFID tag was 2$T_{h}$, for the RFID reader was 2$T_{h}$, and for the database server was 4$T_{h}$; therefore, the total computational cost in their framework was 8$T_{h}\approx 0.0184$. The protocol presented in [20] required 2$T_{h}$, 2$T_{h}$, and 4$T_{h}+2T_{E/D}$ for each RFID tag, RFID reader, and database server, respectively, and its total computational cost was 8$T_{h}+2T_{E/D}\approx 0.0276$.

**Figure 2:**
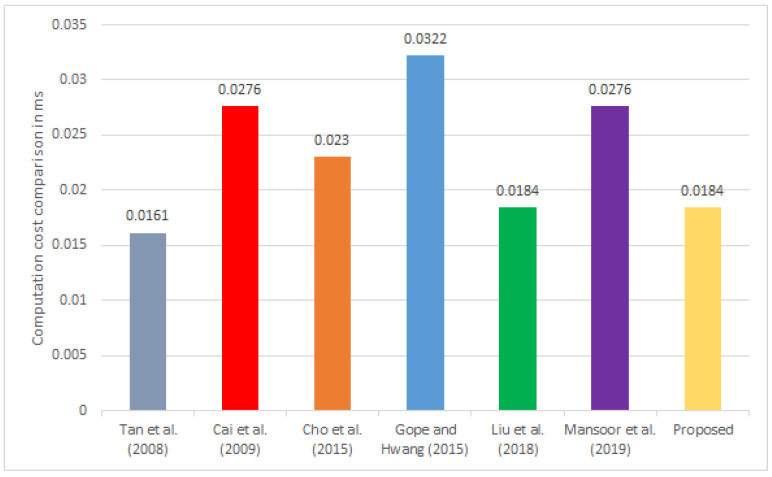
Comparison of the computational cost.

Furthermore, we computed the computational cost of the proposed framework, which required $2T_{h}+T_{E/D}$ for the RFID tag and for the database side $2T_{h}+T_{E/D}$; thus, the total computational cost of the operations of the proposed framework was $4T_{h}+2T_{E/D}\approx 0.0184$. The results based on the comparison given in Table 9 are also visualized in Figure 2. 

### 5.3. Communication Cost Comparison

**Figure 3:**
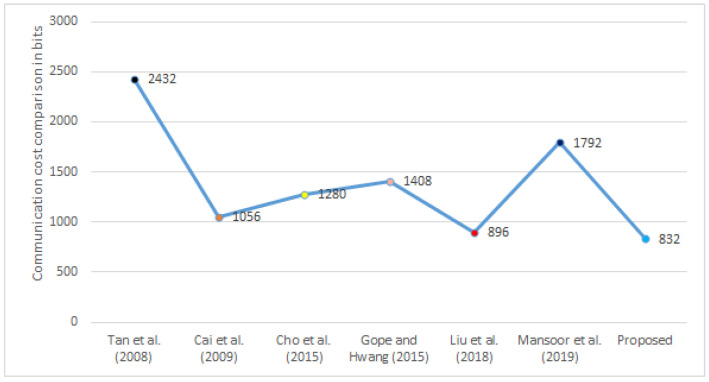
Comparison of the computation cost.

In Table 10, we compute the communication cost of our proposed protocol and other existing protocols [13,16,17,19,20,21]. After that, in Figure 3, we compare the communication costs of the proposed framework to those of different frameworks in the same environment. This demonstrates that the suggested framework has less communication cost than alternative frameworks [13,16,17,19,20,21]. Furthermore, we computed the communication cost of every framework as under a random number, timestamp, and identity taking 64 bits. Here, we used 160 bits for the hash function message digest (SHA-1) and 256 bits for symmetric key encryption/decryption (AES-256).

### 5.4. Conclusions

In this paper, we proposed a unique hash-based lightweight authentication framework for IoT-based RFID communication environments, after a thorough examination of the various types of RFID authentication and key agreement protocols and their benefits and drawbacks. For secure authentication between valid participants, the protocol uses a hash function and the XoR operations mechanism. We were able to minimize the computational cost of the authentication process by using this technique. When we compared it to other current protocols, our proposed protocol provided improved security while consuming less communication, computational, and storage resources. In the future, the suggested framework could be used in IoT applications such as medical privacy protection, the Internet of Vehicles (IoV), smart city environments, and healthcare systems. 

## Figures and Tables

**Table 1:** IoT-based RFID tag features’ comparison.

Features	Active Tags	Passive Tags
Data Storage	128 bytes	128 bytes
Tag Battery	Yes	No
Range	Up to 100 M	Up to 3–5 M
Multiple Tag Reading	More then 1000 tags recognized up to 100 mph	Less than a thousand tags within 3 M of the reader’s range
Signal Strength Required to Tag	Very low	Very high
Tag Power	Internal source to tag	Energy transferred through radio frequency from the reader
Availability of Source Power	Continuous	Only in range of radar

**Table 3:** Notations.

Symbol	Description
$T_{i}$	*i*th RFID tag
$R_{j}$	*j*th RFID reader
⊕	Bitwise XoR operation
h(·)	Cryptographic one-way hash function
$x_{S}$	Secret key of *S*
*S*	Database server
▵T	Maximum time delay in communication
‖	Concatenation operation
$SK_{ij}\left(.\right)$	Session key agreement between entities *i* and *j*
$i\overset{?}{=}j$	Whether *i* equals *j*
A	Adversary
≈	Approximate value
$ID_{T_{i}}$	The identity of the *i*th tag
i···⇒j:{M}	*i* sends message *M* to *j* via a secure channel
i···→j:{M}	*i* sends message *M* to *j* via a public channel

**Table 4:** Registration phase of RFID tag.

Tag $T_{i}$	Database Server *S*
Inputs $ID_{T_{i}}$	
Sends $M_{R_{i1}}=\left\{ID_{T_{i}}\right\}$	
················⇒	Generates sequence number $SN_{i}$ for $T_{i}$
	Computes $S_{1}=ID_{S}⊕h(ID_{T_{i}}∥SN_{i}∥x_{S})$
	Where $x_{S}$ is the private key of *S*
	Computes $S_{2}=h\left(S_{1}∥ID_{T_{i}}\right)⊕ID_{T_{i}}$
	Stores $S_{1},S_{2},SN_{i}$ in the database
	Sends $M_{R2_{i2}}=\left\{S_{1},S_{2},SN_{i},h\left(.\right)\right\}$
upon receiving $M_{R2_{i2}}$	⇐················
Stores $\left\{S_{1},S_{2},SN_{i}\right\}$ in the database	

**Table 5:** Login and authentication phase of RFID.

RFID Tag $T_{i}$	RFID Reader $R_{j}$	Database Server *S*
Generates random value *r*		
Computes $r_{1}=r⊕\left(S_{1}⊕S_{2}\right)$		
Computes $H_{1}=h(ID_{T_{i}}∥S_{1}∥S_{2})$		
Computes $H_{1}^{1}=H_{1}⊕S_{2}$		
Sends $M_{1}=\left\{r_{1},H_{1}^{1},T_{1}\right\}$		
················→	Verifies $T_{2}-T_{1}\leq ▵T$	
	Sends $M_{2}=\left\{r_{1},H_{1}^{1},T_{3}\right\}$	
	················→	Verifies $T_{4}-T_{3}\leq ▵T$
		Computes $H_{1}^{*}=H_{1}⊕S_{2}$
		Verifies $H_{1}^{*}\overset{?}{=}H_{1}^{1}$
		Computes $r^{*}=r_{1}⊕\left(S_{1}⊕S_{2}\right)$
		Generates random value $r_{2}$
		Computes $SK_{S}=h(ID_{S}∥ID_{T_{i}}∥r^{*}∥$
		$r_{2}∥SN_{i}∥S_{1}∥S_{2}∥T_{5})$
		Computes $H_{2}=h(S_{1}∥S_{2}∥r^{*})$
		Computes $H_{2}^{2}=H_{2}⊕\left(r^{*}⊕S_{2}\right)$
		Computes $K_{1}=ID_{T_{i}}⊕h(r^{*}∥SN_{i}∥H_{1}^{*})$
		Encrypts $E_{1}=E_{K1}\left(H_{2}^{2},r_{2},ID_{S},T_{5}\right)$
		Sends $M_{3}=\left\{E_{1},T_{5}\right\}$
		←················
	Verifies $T_{6}-T_{5}\leq ▵T$	
	Sends $M_{4}=\left\{M_{3},T_{7}\right\}$	
	←················	
Verifies $T_{8}-T_{7}\leq ▵T$		
Computes $K_{2}=ID_{T_{i}}⊕h(r∥SN_{i}∥H_{1})$		
Decrypts $\left(H_{2}^{2},r_{2},ID_{S},T_{5}\right)=D_{K2}\left(E_{1}\right)$		
Computes $H_{2}^{*}=H_{2}^{2}⊕\left(r⊕S_{2}\right)$		
Verifies $H_{2}^{*}\overset{?}{=}H_{2}$		
Computes $SK_{T}=h(ID_{S}∥ID_{T_{i}}∥r∥r_{2}∥SN_{i}∥S_{1}∥S_{2}∥T_{5})$		

**Table 6:** Challenge: handshake authentication for the RAFI.

RFID Tag $T_{i}$	RFID Reader $R_{j}$	Database Server *S*
Challenge		
················→		
	Challenge	
	················→	
		Response
		←················
	Success then	
	Response	
	←················	
Success		

**Table 8:** Comparison security and functionality features.

Security Features	[16]	[17]	[13]	[21]	[19]	[20]	Proposed
$RAFI_{1}$	×	✓	✓	✓	✓	✓	✓
$RAFI_{2}$	×	×	✓	✓	✓	×	✓
$RAFI_{3}$	×	✓	×	✓	✓	×	✓
$RAFI_{4}$	✓	×	✓	✓	×	×	✓
$RAFI_{5}$	×	×	×	✓	×	✓	✓
$RAFI_{6}$	×	×	✓	×	×	×	✓
$RAFI_{7}$	×	×	✓	×	×	×	✓
$RAFI_{8}$	×	×	×	✓	✓	×	✓
$RAFI_{9}$	✓	✓	✓	×	×	✓	✓
$RAFI_{10}$	✓	×	✓	✓	✓	✓	✓
$RAFI_{11}$	×	×	×	✓	✓	×	✓
$RAFI_{12}$	✓	×	×	×	✓	×	✓
$RAFI_{13}$	×	✓	✓	✓	✓	×	✓
$RAFI_{14}$	×	×	×	✓	×	✓	✓
$RAFI_{15}$	×	×	×	✓	✓	×	✓

Note ⇒ ×: not secure against the attack; ✓: secure against the attack; “*RAFI*_1_: mutual authentication; *RAFI*_2_: tag untraceability; *RAFI*_3_: tag anonymity; *RAFI*_4_: backward/forward secrecy; *RAFI*_5_: scalability; *RAFI*_6_: collision attacks; *RAFI*_7_: dos attacks; *RAFI*_8_: replay attacks; *RAFI*_9_: stolen verifier attacks; *RAFI*_10_: de-synchronization attacks; *RAFI*_11_: man-in-the-middle attack; *RAFI*_12_: impersonation attack; *RAFI*_13_: message authentication; *RAFI*_14_: data confidentiality; *RAFI*_15_: insider attack”.

**Table 9:** Comparison of the computational cost.

	Tag	Reader	Server	Total Operations	Execution Cost (ms)
[16]	$2*T_{h}$	$2*T_{h}$	3$*T_{h}$	$4*T_{h}$	0.0161
[17]	$4*T_{h}$	$2*T_{h}$	$6*T_{h}$	$12*T_{h}$	0.0276
[13]	3$*T_{h}$	$2*T_{h}$	$5*T_{h}$	$10*T_{h}$	0.023
[21]	$5*T_{h}$	$2*T_{h}$	$7*T_{h}$	$14*T_{h}$	0.0322
[19]	$2*T_{h}$	$2*T_{h}$	$4*T_{h}$	$8*T_{h}$	0.0184
[20]	$2*T_{h}$	$2*T_{h}$	$4*T_{h}+2*T_{E/D}$	$8*T_{h}+2*T_{E/D}$	0.0276
Proposed	$2*T_{h}+T_{E/D}$	−	$2*T_{h}+T_{E/D}$	$4*T_{h}+2*T_{E/D}$	0.0184

**Table 10:** Communication cost comparison with relevant frameworks.

	Communication Costs in Bits	No. of Messages
[16]	2432	4
[17]	1056	5
[13]	1280	5
[21]	1408	4
[19]	896	4
[20]	1792	4
Proposed	832	4

## Data Availability

Not applicable.
